# A Case Report of a Migrated Pelvic Coil Causing Pulmonary Infarct in an Adult Female

**DOI:** 10.5811/cpcem.2020.5.47463

**Published:** 2020-07-09

**Authors:** Angel Guerrero, Rebecca G. Theophanous

**Affiliations:** Duke University Medical Center, Department of Surgery, Division of Emergency Medicine, Durham, North Carolina

**Keywords:** Migrated coil, pelvic congestion syndrome, pulmonary infarct

## Abstract

**Introduction:**

It is possible but rare for a pelvic coil to migrate to the pulmonary vasculature, which can cause cardiac damage, arrhythmias, pulmonary infarct, and thrombophlebitis. The few cases reported typically do not describe removal of the coils, as patients were asymptomatic.

**Case report:**

A 39-year-old female with recent coil embolization of her left internal iliac and ovarian veins for pelvic congestion syndrome presented with one month of right-sided chest pain and dyspnea. Imaging revealed a migrated pelvic coil in the patient’s right main pulmonary artery with pulmonary infarcts and a pleural effusion.

**Conclusion:**

Interventional radiology successfully removed the coil endovascularly, with significant symptom improvement. This prevented a more-invasive open surgical procedure and resolved symptoms without requiring long-term anticoagulation or monitoring.

## INTRODUCTION

Pelvic congestion syndrome is chronic pelvic pain caused by gonadal vein varicosities, worsened by prolonged standing, sexual intercourse, menstruation, or pregnancy. Multiparous women of reproductive age are at increased risk, and the overall prevalence is about 30%.^1^ Venogram is the diagnostic gold standard, and potential treatments include hormone therapy, vasoconstrictive medications, or pelvic coil embolization.^1^,^2^ It is possible but rare for a pelvic coil to migrate to the pulmonary vasculature, and the few cases reported typically do not describe removal of the coils, as patients were asymptomatic.^3^,^4^ We present an adult female with pelvic congestion syndrome status post coil embolization with chest pain and dyspnea, found to have a migrated pelvic coil in her right pulmonary artery.

## CASE REPORT

A 39-year-old female with pelvic congestion syndrome had undergone coil embolization of the left internal iliac and ovarian veins three months earlier. For the prior month, the patient endorsed right-sided pleuritic chest pain and dyspnea with orthopnea. Differential diagnosis included a viral or bacterial pulmonary infection, pulmonary embolism, acute coronary syndrome, pericarditis, and less likely pneumothorax, aortic dissection, or congestive heart failure. Outpatient chest radiograph (CXR) demonstrated an ectopic coil in the right pulmonary vasculature, with a second coil still in place in the left ovarian vein on subsequent abdominal radiograph.

**Image 1:**
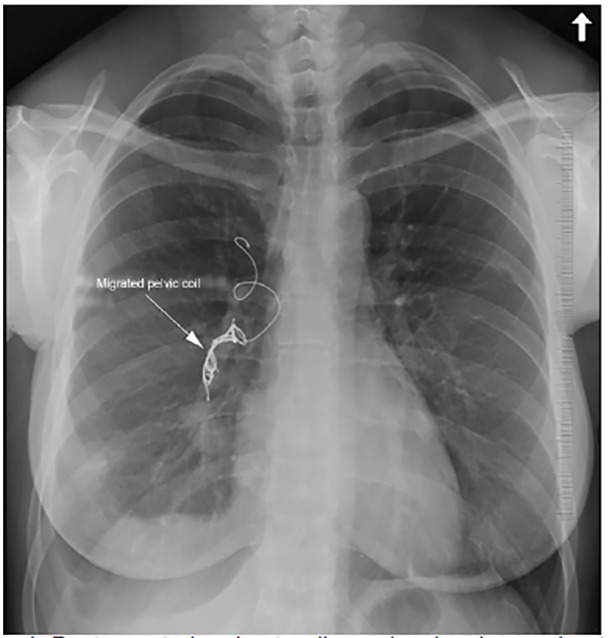
Posteroanterior chest radiography showing a migrated pelvic coil (arrow) in the right pulmonary artery extending into segmental branches.

On emergency department evaluation, the patient had normal vitals including 100% oxygen saturation on room air with a respiratory rate of 18 breaths per minute, clear breath sounds, and no leg edema. Labs were unremarkable. Electrocardiogram demonstrated normal sinus rhythm at 84 beats per minute. CXR and computed tomography (CT) imaging revealed an ectopic pelvic coil in the right main pulmonary artery extending into multiple upper and lower lobe segmental branches (Image 1).

**Image 2:**
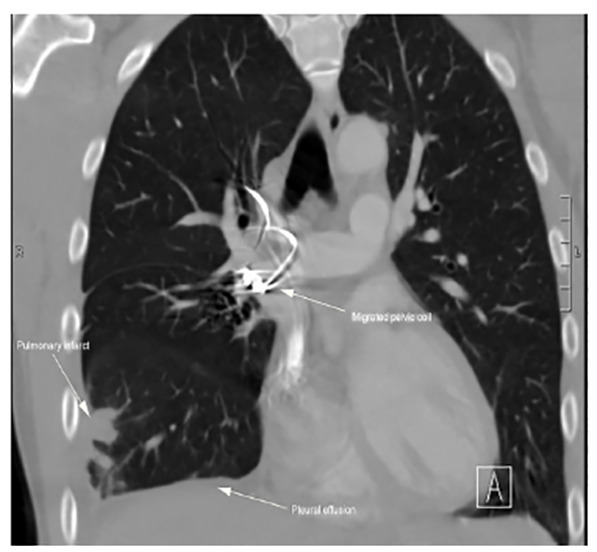
Computed tomography imaging showing a migrated pelvic coil (mid-image arrow) in the right pulmonary artery with areas of pulmonary infarct in the right middle and lower lobes and a small right pleural effusion (left and lower arrows).

Coil artifact somewhat limited the identification of thrombus, but there were peripheral wedge-shaped opacities in the right middle and lower lobes concerning for infarcts and a small right pleural effusion (Image 2). There was no evidence of right heart strain on CT.

Following consultation with vascular surgery, it was decided that vascular interventional radiology (VIR) would be the least invasive yet most likely successful method for coil retrieval when compared to an open surgical approach. The patient was consented and transported directly to VIR, where the groin was prepped in standard fashion. The right common femoral vein was accessed with a micropuncture kit using ultrasound guidance. A pulmonary angiography catheter was advanced over a guidewire into the right main pulmonary artery via a 7 French sheath. Contrast phase did not show significant clot within the artery.

**Image 3:**
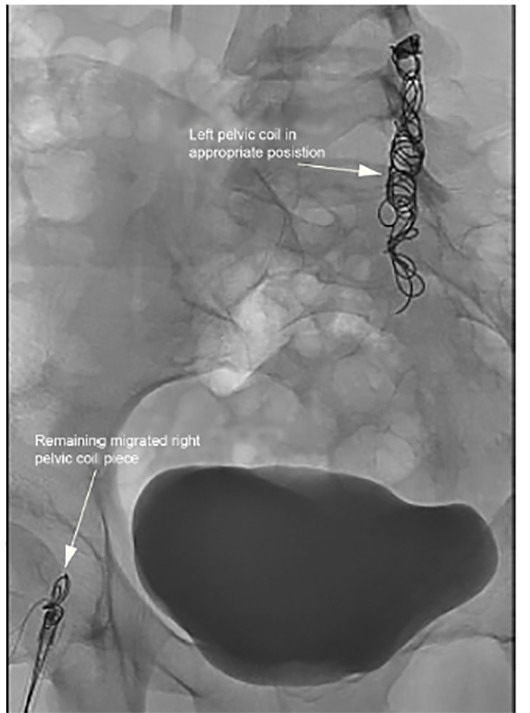
Fluoroscopy demonstrating a piece of migrated pelvic coil from the right lung now in the right femoral vein (lower arrow), as well as the remaining pelvic coil in place in the left ovarian vein (upper arrow).

The sheath was exchanged for a long 7 French sheath with the tip in the right pulmonary artery. Multiple snares were passed through the sheath to engage the 20-millimeter (mm) Nester coil pack (Cook Medical, Bloomington, IN); however, the coil unraveled into small pieces, until eventually a large piece was snared and retracted to the right femoral vein. Interventional radiology then performed en bloc removal through the right groin access site given the coil was too large to pass through the sheath; however, a piece of coil remained in the right femoral vein (Image 3). Multiple attempts to snare the coil via an upsized 11 French sheath were still unsuccessful.

Similarly, the left femoral vein was accessed and upsized to a 9 French sheath, which finally allowed for successful snare removal of the remaining coil. Repeat imaging showed a small residual coil fragment in the right mid-lung that was deemed not to cause increased injury, thus was left in situ. An intact, 16mm Nester coil pack was noted in the left gonadal vein.

**CPC-EM Capsule**

What do we already know about this clinical entity?

The migration of endovascular coils is a relatively rare complication, with few cases reported in patients with pelvic congestion syndrome.

What makes this presentation of disease reportable?

Endovascular retrieval is a less invasive treatment modality compared to surgery with fewer risks and decreased recovery time.

What is the major learning point?

Emergency clinicians should be able to recognize, stabilize, and initiate treatment for complications of surgical procedures including coil embolization.

How might this improve emergency medicine practice?

Endovascular retrieval can be a safe intervention, resulting in symptom resolution without the requirement of long-term anticoagulation or monitoring.

The patient was observed in the hospital overnight and went home the following day without anticoagulation or other acute complications. The patient followed up with vascular surgery clinic several months later for recommendations on her remaining gonadal vein coil with no additional interventions.

## DISCUSSION

Coil embolization has been used since demonstrating efficacy in arterial occlusion in 1975 and in the treatment of pelvic congestion syndrome since first described by Edwards et al in 1993.^2^,^3^ Current literature reports high symptomatic improvement rates of 70–85% for percutaneous vein embolization, whereas pharmacotherapy has had poor success in achieving pain relief.^3^ Surgical ligation is another effective treatment but is much more invasive.^1^

Complications of pelvic embolization include vein perforation and coil migration (either immediate or delayed) causing cardiac damage, arrhythmias, pulmonary infarct, and thrombophlebitis, with rates ranging from 4–8%.^4^–^6^ Few cases have been reported regarding migration of pelvic coils to the pulmonary vasculature, and rarely with symptomatic patients requiring endovascular retrieval of the coils.^7^,^8^

Yamasaki et al described migration of nine internal iliac vein coils to the pulmonary artery, but the coils were not removed as the patient was asymptomatic. They, along with other studies, postulated that coils should be at least 30–50% the diameter of the target vessel in order to decrease migration risk. They also describe using coils with a stronger radial force, for example measuring 0.035 inch, as veins have lower frictional resistance (increased elasticity) between the vessel wall and the coils. Furthermore, larger vessels with a high-flow state (such as the internal iliac, as in our patient), are at higher risk for coil migration, especially when the varices are relieved and flow is increased.^9^ Tonkin et al described two cases of coil migration to the tricuspid valve and pulmonary arteries with a coil fragment in the right ventricle, which were asymptomatic and conservatively managed.^10^

None of these cases include removal of the coils nor the methods behind the retrieval process. Our patient developed pulmonary infarcts and a pleural effusion, which has not been previously reported, and thus necessitated urgent removal of the migrated coil, as we have described above. Although there were difficulties with VIR removal of the coil, this still prevented the patient from undergoing an open surgical procedure, which could have led to prolonged recovery time, longer hospital stay, and other post-operative complications such as non-healing wounds, infection, hemorrhage, pulmonary embolism, etc. Our patient had complete resolution of her symptoms and no additional complications on follow-up.

## CONCLUSION

The migration of endovascular coils is a relatively rare complication, with few cases reported in patients with pelvic congestion syndrome. Additionally, the coil retrieval process has not been well described. Careful history should be obtained in patients presenting with chest pain or shortness of breath, including recent procedures. Plain film imaging is a rapid and useful tool to easily assess for coil migration. Endovascular retrieval of the migrated coil was a successful and safe intervention in this patient, resulting in symptom resolution without the requirement of long-term anticoagulation or monitoring.
